# Supplementary material for: Direction-dependent dynamics of colloidal particle pairs and the Stokes-Einstein relation in quasi-two-dimensional fluids
Source: Nat Commun. 2023 Aug 22;14:5109. doi: 10.1038/s41467-023-40772-2 (PMC10444761; doi:10.1038/s41467-023-40772-2)
Supplement: Supplementary file 1 — Supplementary Information [file 41467_2023_40772_MOESM1_ESM.pdf]

Supplementary Information for "Direction-dependent Dynamics of Colloidal Particle Pairs and the Stokes-Einstein Relation in Quasi-Two-Dimensional Fluids"

Noman Hanif Barbhuiya[1], A. G. Yodh[2] and Chandan K. Mishra[1*]

[1]Discipline of Physics, Indian Institute of Technology Gandhinagar, Palaj, Gandhinagar, 382055, Gujarat, India.
[2]Department of Physics and Astronomy, University of Pennsylvania, Philadelphia, 19104, Pennsylvania, USA.

*Corresponding author(s). E-mail(s): chandan.mishra@iitgn.ac.in;
Contributing authors: barbhuiyanoman@iitgn.ac.in;
yodh@physics.upenn.edu;

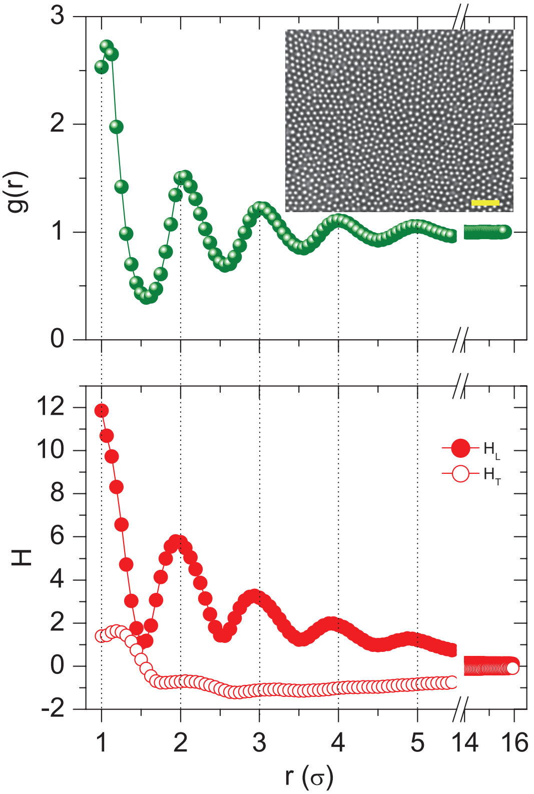

Fig. S1: Connection between hydrodynamics and underlying structure of the fluid. a, The sample pair correlation function, $g(r)$, with r expressed in units of particle diameter (σ), at $\phi = 0.61$. Inset: one quarter of a typical field-of-view of the colloidal fluid at $\phi = 0.61$ with scale bar of 5 μm. **b,** H_L (solid red), H_T (open red) evaluated for $t = 0.5$ s versus r for quasi-two-dimensional colloidal fluids at $\phi = 0.61$. The vertical lines show that the peaks of $g(r)$ and $H_L(r)$ coincide.

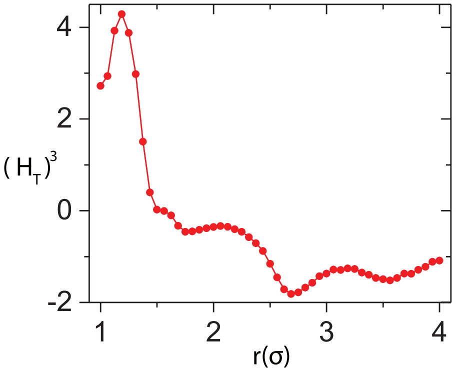

Fig. S2: Revealing spatial positions of maxima and minima in H_T. $(H_T)^3$ versus $r(\sigma)$ at $\phi = 0.61$ confirming spatial phase lag of 0.25σ between H_L and H_T. While H_L peaks at $r = \{1, 2, 3, ...\}\sigma$, H_T peaks at $r = \{1.25, 2.25, 3.25, ...\}\sigma$.

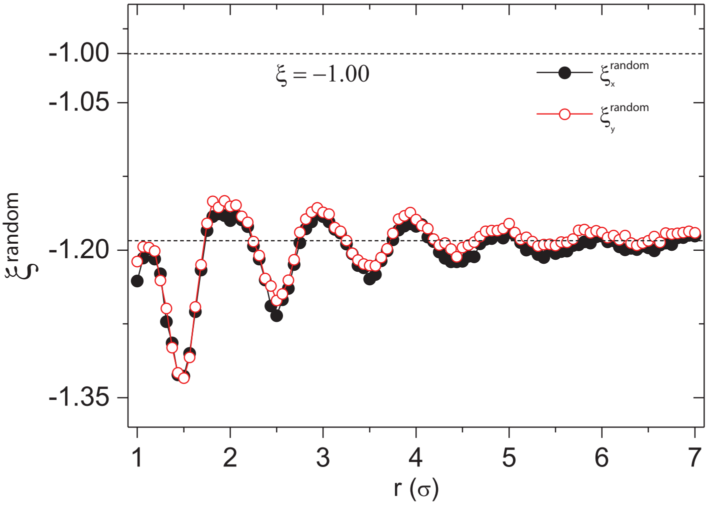

Fig. S3: Stokes-Einstein exponent measured for displacements in random (lab frame) directions. We measured displacement particles in particle-pairs along two randomly chosen orthogonal directions, x and y, in the lab frame. Then we followed the same prescription as described in the main text for the L and T directions to measure the Stokes-Einstein exponent, ξ, along the x and y directions. Notice that the resulting exponents, along randomly chosen directions, vary spatially with r but are *not anisotropic*.

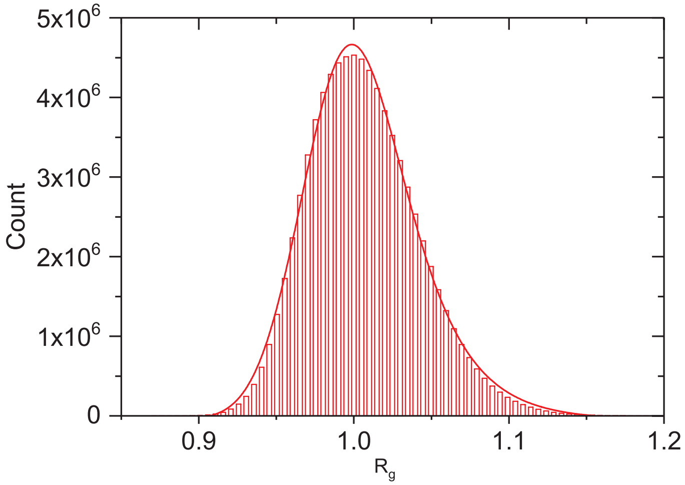

Fig. S4: Ensuring quasi-2D nature of the experimental cell. Histogram of the apparent diameter of the particles, $\sigma^{apparent}$, measured from spatial intensity distribution of the tracked particles in the field-of-view from all the images at $\phi = 0.61$. $\sigma^{apparent}$ is normalized by its most-probable value. The solid line is Gaussian fit to the distribution. The polydispersity in $\sigma^{apparent}$, and hence, the size of the particles measured from the distribution $\sim 3\%$, which is comparable to the size polydispersity quoted by the manufacturer ($< 5\%$). This confirms that particles are indeed confined to a quasi-2D plane over the experimental field-of-view. Note, if the height of the cell was larger than the particle diameter, a significant variation in the intensity distribution of particles, and hence, their $\sigma^{apparent}$ due to the freedom to move out of the focal plane would have been observed. Note, the measured polydispersity could be due to either size polydispersity or height variation or a combination thereof.

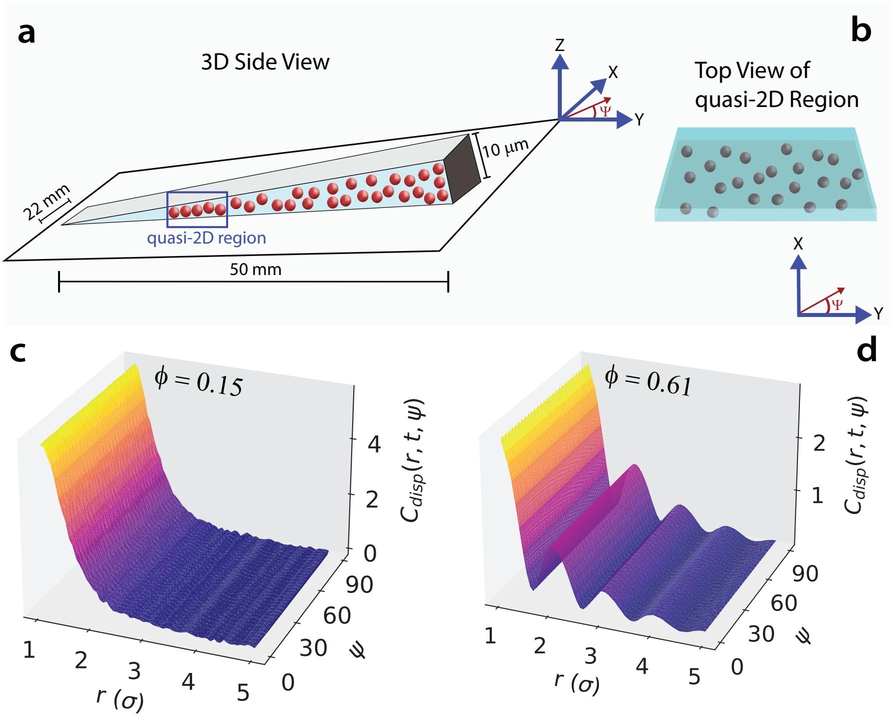

Fig. S5: Schematic of the experimental cell and displacement correlation between colloids in colloid-pair in the lab frame. Schematic of **a,** 3D side and **b,** top quasi-2D region view of the experimental wedge-shaped cell with the wedge along the $y-$axis in the lab frame. To ensure that choice of the experimental cell does not influence the dynamics measurement of colloids in colloid-pair, we have computed displacement correlation function $C_{disp}(r,t)$ at various angle ψ with respect to the wedge-direction ($y-$axis in the lab frame). We define $C_{disp}(r,t,\psi) = \langle \Delta\mathbf{r}_i^{\psi}(\mathbf{r}',t).\Delta\mathbf{r}_j^{\psi}(\mathbf{r}'+\mathbf{r},t)\rangle$. Here, $\Delta\mathbf{r}_i^{\psi}$ are the displacements of ith particle resolved in a direction making an angle ψ with the y-direction in the lab frame. The three-axes plot of $C_{disp}(r,t,\psi)$ at **c,** $\phi = 0.15$ and **d,** $\phi = 0.61$. Absence of any variation in $C_{disp}(r,t)$ with ψ suggests dynamics revealed in the main text for colloids in colloid-pairs body-frame are due to unique features of hydrodynamic interactions in quasi-2D confinement and rules out its dependence on the choice of the shape of the experimental cell.

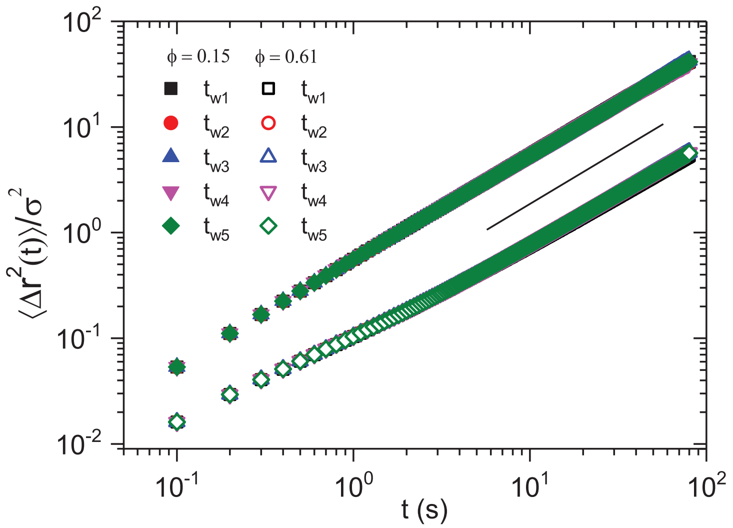

Fig. S6: Ensuring equilibration of samples. To check that colloidal fluids at all ϕs have equilibrated, the video microscopy data at each ϕ for entire experimental duration was split into 5 equal non-overlapping time windows, t_{w1} to t_{w5}. The plot shows mean-squared displacement $\Delta r^2(t)$ versus t for each of these time windows at $\phi = 0.15$ (solid symbols) and at $\phi = 0.61$ (open symbols). Clearly, the dynamics is not dependent on the time-windows chosen and hence the colloidal fluid at each particle packing area fraction has equilibrated.

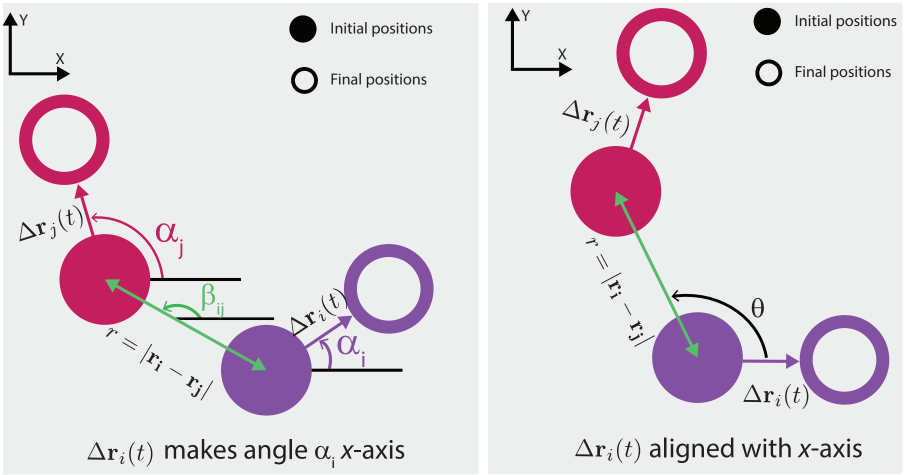

Fig. S7: Schematic for evaluating hydrodynamic displacement flow field. Consider a pair of particles, $\{i, j\}$. $\Delta\mathbf{r}_i(t = 0.5\text{s})$, $\Delta\mathbf{r}_j(t = 0.5\text{s})$, and the line joining i to j subtend angles α_i, α_j, and β_{ij} with respect to the positive $x-$axis (left image). To derive the plot shown in Figure 1f, we rotate the 2D coordinate system through an angle α_i for each reference particle i, so that $\Delta\mathbf{r}_i(t = 0.5\text{s})$ is aligned along the positive x (horizontal) direction. The position coordinate of the $j^{th}-$ particle is now defined as $\mathbf{r}(r, \theta)$ with $i^{th}-$particle as reference (right image).

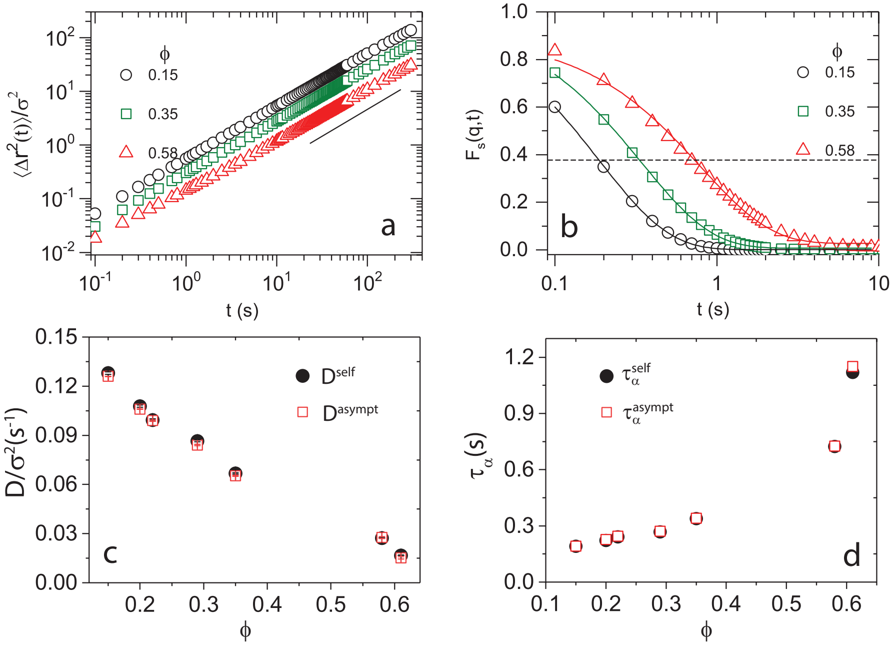

Fig. S8: Dynamics measurements. a, Mean squared displacement, $\langle \Delta r^2(t)\rangle$, and the **b,** self intermediate scattering function, $F_s(q,t)$, versus lag time t for particles in the samples at different packing area-fraction. Distinct colors (and shapes) represent different ϕ. D^{self} is measured from the linear regime in **a** shown by the solid line with slope 1. The solid lines in **b** are guide to the eye; relaxation time τ_α^{self} is defined from $F_s(q, t = \tau_\alpha^{self}) = 1/e$ (see horizontal dashed line). **c,** Comparison of D^{self} obtained from the data above (black symbols) with asymptomatic values derived in the same samples from the body frame analysis (red symbols), *i.e.*, D^{asympt} $(= (D^L + D^T)$ for $r > 8\sigma)$ as a function of ϕ. The errors bars in D are from fittings. **c,** Analogous comparison of τ_α^{self} (black symbols) with asymptomatic values obtained from the body frame (red symbols) τ_α^{asympt} $(= (\tau_\alpha^L + \tau_\alpha^T)/2$ for $r > 8\sigma)$ with ϕ.

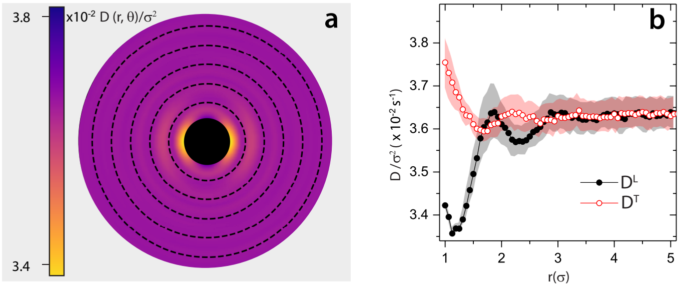

Fig. S9: Spatially inhomogeneous and anisotropic diffusivity at $\phi = 0.35$, a Polar colormaps $D(r,\theta)$ versus r. The dashed radial circles are at $r = \{2, 3, 4, ...\}\sigma$. **b,** $D(r)$ along L and T directions corresponding to $\theta = 0°$ and $\theta = 90°$, respectively. The error bars in D are from fittings.
