# Supplementary material for: Direction-dependent dynamics of colloidal particle pairs and the Stokes-Einstein relation in quasi-two-dimensional fluids
Source: Nat Commun. 2023 Aug 22;14:5109. doi: 10.1038/s41467-023-40772-2 (PMC10444761; doi:10.1038/s41467-023-40772-2)
Supplement: Supplementary file 3 — Description of Additional Supplementary Files [file 41467_2023_40772_MOESM3_ESM.pdf]

Description of Additional Supplementary Files

File Name: Supplementary Video 1

Conditional $P(\Delta\mathbf{r}(t))$ measured for the particle on the right of the pair ($r = 2.0$ σ) when the particle on the left moves (is displaced) along the L direction during a time lag $t = 0.5$ s at $\phi = 0.15$. The video reveals longitudinal drag motion. The solid red and white circles represent the mean positions of the particles at t_0 and $(t_0 + t)$, respectively.

File Name: Supplementary Video 2

Conditional $P(\Delta\mathbf{r}(t))$ measured for the particle on the right of the pair ($r = 2.5$ σ) when the particle on the left moves (is displaced) along the T direction during a time lag $t = 0.5$ s at $\phi = 0.15$. The video reveals transverse anti-drag motion. The solid red and white circles represent the mean positions of the particles at t_0 and $(t_0 + t)$, respectively.

File Name: Supplementary Video 3

Conditional $P(\Delta\mathbf{r}(t))$ measured for the particle on the right of the pair ($r = 1.1$ σ) when the particle on the left moves (is displaced) along the T direction during a time lag $t = 0.5$ s at $\phi = 0.15$. The video reveals "mass void filling" motion. The solid red and white circles represent the mean positions of the particles at t_0 and $(t_0 + t)$, respectively.
